# Optimization of Methodologies to Study Freeze/Thaw Processes in Drug Substance Bottles

**DOI:** 10.3390/mps7050068

**Published:** 2024-09-04

**Authors:** Sarah S. Peláez, Hanns-Christian Mahler, Jörg Huwyler, Andrea Allmendinger

**Affiliations:** 1 ten23 Health AG, Mattenstrasse 22, 4058 Basel, Switzerland; sarah.pelaez@ten23.health (S.S.P.); hanns-christian.mahler@ten23.health (H.-C.M.); 2 Institute of Pharmaceutical Technology, Goethe University Frankfurt, Max-von-Laue-Strasse 9, 60438 Frankfurt am Main, Germany; 3 Department Pharmaceutical Sciences, University of Basel, Klingelbergstrasse 50, 4056 Basel, Switzerland; joerg.huwyler@unibas.ch; 4 Institute of Pharmaceutical Sciences, Department of Pharmaceutics, University of Freiburg, Sonnenstr. 5, 79104 Freiburg, Germany

**Keywords:** freezing, thawing, drug substance, temperature mapping, liquid sampling, concentration gradient, thawing time, last point to freeze, drug substance bottles, process characterization

## Abstract

Biological drug substance (DS) is often frozen to enhance storage stability, prolong shelf life, and increase flexibility during manufacturing. However, the freezing and thawing (F/T) of bulk DS at the manufacturing scale can impact product quality as a result of various critical conditions, including cryo-concentration during freezing, which are influenced, among other things, by product-independent process parameters (e.g., container type, fill level, F/T equipment, and protocols). In this article, we report the optimization of two major methodologies to study product-independent process parameters in DS bottles at the manufacturing scale, namely the recording of temperature profiles and liquid sampling after thawing to quantify the concentration gradients in the solution. We report experimentally justified measuring positions for temperature recordings, especially for the selection of the last point to freeze position, and highlight the implementation of camera-assisted inspection to determine the last point to thaw and the actual thawing time. In particular, we provide, for the first time, a detailed description of the technical implementation of these two measuring set-ups. Based on the reported case studies, we recommend choosing relevant measuring positions as a result of initial equipment characterization, resulting in a resource-conscious study set-up.

## 1. Introduction

Biological drug substance (DS) is often stored in a frozen state to increase stability and shelf life. However, the freezing and thawing (F/T) of bulk DS at the manufacturing scale are critical unit operations, which can cause protein aggregation and sub-visible particle formation, and thus may impact product quality. A variety of underlying mechanisms that may trigger F/T-related product instabilities have been described. These include, for example, cryo-concentration during freezing [1]; interaction with surfaces like the ice surface [2] or formed air bubbles; [3] and strong concentration gradients in the solution after thawing. Concentration gradients result from the gravitational settling of highly concentrated fractions released during thawing, while the upper region is increasingly diluted by the remaining ice floating on top [4,5].

Several process parameters of the F/T process have been found to contribute to product degradation. These range from container-specific aspects, including type, geometry and fill level, to process-specific aspects, including the number of F/T cycles, F/T equipment, and protocols [6,7,8], which both impact F/T rates and F/T directions [9,10,11]. Process characterization studies are therefore crucial to understand the impact of equipment capabilities and process parameters on the critical conditions for the product.

Temperature mapping during F/T and liquid sampling after one or several F/T cycles are the two most commonly used methodologies to characterize F/T processes at scale and can be also employed to develop and verify novel methodologies like small-scale models or computerized simulation models.

Temperature mapping during F/T is applied to determine characteristic time spans, such as the stress time, freezing time, thawing time, or the overall process time, as well as other parameters such as cooling/heating rates or the freeze front velocity. For example, the stress time [12], which has also previously been referred to as residence time [13], describes the time a product remains in the partially frozen state during freezing, and is quantified as the time between the onset of nucleation until reaching the glass transition temperature at the last point in the bottle. The thawing time is an important characterization parameter, as slow thawing, for example, is generally considered critical for protein stability compared to fast thawing [8,14]. Such parameters allow for a quantitative comparison of process conditions, especially those critical for product stability.

In order to define the relevant time spans based on temperature data, it has been previously suggested to select the last point to freeze (LPF) and the last point to thaw (LPT) in a DS container [15]. However, it is not obvious where these specific points are located. The LPF position, for example, is dependent on container-specific factors like the container geometry, size, and fill volume, and also on process-specific factors like freezing point depression, convection in the unfrozen solution, and the freezing front direction including the formation of ice mounds [16,17,18]. Most importantly, the selection of the LPF position has, in many cases, not been experimentally informed, or it has not been described how it was identified. Assumptions for the LPF vary widely from the geometrical center of the liquid [19], to a few centimeters below the liquid level in the center [12,20], or at the liquid level in the center [15]. In addition to the determination of the LPF and LPT, a temperature probe at the FPF position has typically also not been included in previous studies, even though the stress time is based on the time point when freezing in the container starts, which is the onset of ice nucleation. The position of the LPT is container and process specific. In addition, it is also subject to the phenomenon of the ice detaching from the temperature probes at a certain point in time, which inevitably affects the measurements. In previous studies, this phenomenon has been largely disregarded in the evaluation of thawing times, and only a few authors have mentioned it [21,22]. A practical approach to account for this bias is lacking and it is currently addressed by visual observation and documentation by an operator [7], which is highly inconvenient.

Adequate descriptions of technical set-ups that enable the placement of temperature probes in DS bottles are typically not provided [1,7,19,20,23]; only highly specific set-ups that cannot be easily transferred, or set-ups that are not versatile enough to enable placement at any desired position based on container geometry are described [4,24].

Liquid sampling after thawing is a commonly applied technique to evaluate product stability after exposure to one or multiple F/T cycles, or to evaluate the concentration gradient in the solution after thawing (i.e., product or surrogate solution), which relates to process-related stresses. Similar to the temperature mapping approach, sampling procedures of the thawed liquid to precisely determine concentration gradients are rarely described in detail, except for the set-up recently reported by Blümel et al. [24], which we further optimized for application in manufacturing-scale (2 L) DS bottles.

In this article, we report a systematic approach to F/T process characterization, using optimized procedures of commonly employed methodologies of temperature mapping and liquid sampling after thawing I and including detailed experimental description. First, we describe an experimental approach to identify and select the relevant temperature probe positions required for the evaluation of the freezing process (i.e., FPF and LPF) for any specific DS container and process. We present a case study using 2 L and 5 L DS bottles filled with a surrogate solution subjected to passive freezing in convectional −80 °C or −40 °C freezers. We further propose a camera-assisted methodology to investigate the generally neglected measurement bias during the thawing procedures of ice detachment from the temperature probes. Next, we describe an optimized methodology for liquid sampling after thawing, which was as investigated in a case study of a surrogate solution from −40 °C to room temperature, and which is applicable for any type of DS bottle.

## 2. Materials and Methods

### 2.1. Materials

An aqueous surrogate formulation representing a typical monoclonal antibody formulation was prepared with 20 mM L-histidine/L-histidine HCl (J.T. Baker, Dietikon, Switzerland), 240 mM sucrose (Pfanstiehl, Zug, Switzerland), 10 mM L-methionine (J.T. Baker), and 0.04% polysorbate 80 (J.T. Baker) at a pH of 5.5. The solution was sterile filtered with a 0.22 µm PVDF filter cup (VWR). Chemicals from J.T. Baker and sterile filters were purchased from VWR (Dietikon, Switzerland), and 2 L and 5 L polycarbonate PharmaTainer^TM^ bottles were provided by SaniSure (Bascharage, Luxembourg).

### 2.2. Freezing/Thawing

Then, 2 L DS bottles were filled with 2 L surrogate solution and frozen to −40 °C or −80 °C using static freezers: a −40 °C low temperature freezer from Snijders Labs (Tilburg, The Netherlands); and a −80 °C ultra-low temperature (ULT) freezer from Thermo Fisher Scientific, (Asheville, NC, USA). One DS bottle at a time was placed in the empty freezer in the center of the lowest shelf and consistent door-opening times of 60 s were applied before starting an experimental run. For thawing, the DS bottles were placed on a planar work bench and left at room temperature (RT) for passive thawing.

Temperature mapping experiments and time-lapse camera monitoring of the DS bottles during thawing were performed with bottles subjected to freezing in the −80 °C freezer. Liquid sampling was performed after freezing to −40 °C and subsequent thawing at RT.

### 2.3. Temperature Mapping Equipment

Typ-T thermocouples (TC) of 1.5 mm diameter from TC GmbH (Mönchengladbach, Germany) were used for temperature measurement inside the DS bottle, and a Pt-100 resistance temperature detector (RTD) from Testo (Mönchaltdorf, Switzerland) was used for temperature measurement outside of the DS bottles. The temperature profiles were recorded using RDXL6SD-USB data loggers from OMEGA Engineering GmbH (Deckenpfronn, Germany) with a measurement interval set to 15 s.

**Figure 1:**
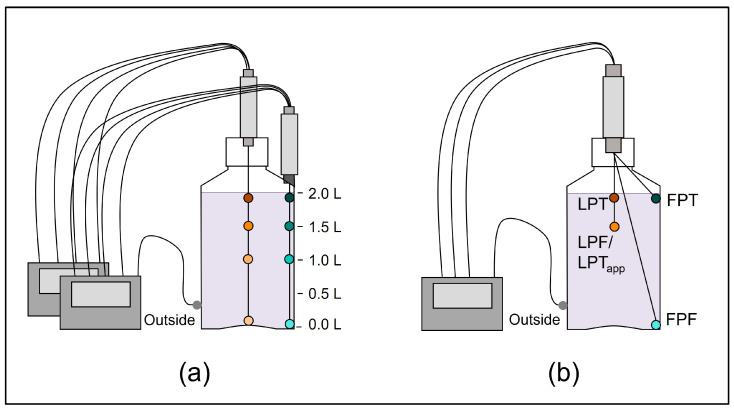
(**a**) Initial temperature mapping with eight temperature probes, four along the center axis (dots with different shades of orange), four along the edge axis (dots with different shades of cyan) and one outside of the bottle (gray) to identify the relevant temperature probe positions. (**b**) Optimized temperature mapping set-up with the four selected positions reflecting the first point to freeze (FPF); the last point to freeze (LPF), which is equal to the apparent last point to thaw (LPT_app_); the first point to thaw (FPT); and a position at the top in the center, close to the expected LPT position due to the floating of ice.

For precise placement of the TCs at defined positions inside the DS bottle, two cable glands from Distrelec AG (Nänikon, Switzerland) were screw connected with a connection pipe with both bottom sides facing each other, while the lower one was fixed through a hole made in the lid (Figure 1). The TCs were then passed through the fixture connected to the lid and arranged and bent into shape if required (Figure 1b) by using a true-scale technical drawing of the DS bottle as a template. The well-arranged TCs in the fixture were placed inside the DS bottle, both cable glands were tightened, and the lid was closed. For the TCs positioned along the edge positions (Figure 1a), the lower cable gland was anchored to the bottle using a 2-component epoxy resin. The method variability was evaluated for the optimized set-up in 2 L DS bottles (Figure 1b) by three independent experimental runs and showed low variability as shown in the Supplementary Material (Figure S1).

For time-lapse monitoring of DS bottles during thawing, a Raspberry Pi system including a Raspberry Pi Camera Module 3 (Totonic GmbH, Zurich, Switzerland) was programmed to record a picture every minute.

### 2.4. Liquid Sampling Equipment

For liquid sampling after thawing, a disposable polymeric syringe equipped with a syringe valve, and a 300 mm long 20 G needle from Cadence Science (Cranston, RI, USA), were fixed on a lab stand with a vertically adjustable lab clamp. Sampling was carried out with a long needle connected to a syringe and syringe valve through the bottle opening for the center positions, or by passing the needle through a 1 mm pinhole made in the bottle for the edge positions. At each position, 2.5 mL was carefully aspired with the syringe and the liquid was transferred into sampling tubes while having the valve closed to avoid back flow and mixing of the liquid. In between sampling positions, 0.5 mL was discarded to account for the dead space of the valve and needle (approx. 0.3 mL). The samples in both vertical axes (center or edge) were collected from top to bottom. For thawing, the bottle was placed on a planar work bench and any movement or vibration of the bottle was avoided.

### 2.5. Analytics

Concentration gradients in the thawed surrogate solution were quantified by osmolality measurements. Samples were analyzed in triplicates by freezing point depression methodology using an Osmomat 3000 instrument from Gonotec that was purchased from Haslab GmbH (Biel-Benken, Switzerland).

## 3. Results and Discussion

### 3.1. Time-Resolved Temperature Mapping

Evaluating the F/T process from temperature profiles to determine characteristic time spans (e.g., stress time, freezing time, process time, thawing time, etc.) and other relevant parameters requires placement of temperature probes at appropriate locations.

We used a surrogate solution with typical formulation components for a mAb. mAbs are usually stabilized by sugars such as trehalose or sucrose against F/T-related stress [25,26]. We chose a surrogate solution, i.e., containing sucrose (resulting in a low T_g_’ of −34 °C) in a histidine buffer with the addition of polysorbate 80 and methionine, but without additional excipients that increase viscosity. Thus, the formulation constitutes the worst case for studying freezing in terms of cryo-concentration and stress times [27]. The use of a surrogate solution is particularly relevant to study product-independent parameters, to reduce material consumption.

To accurately study relevant time spans by temperature mapping, the positions for temperature probe placement need to be identified for the specific DS container and freezing process of interest. The technical set-up, which was described in detail above, enables the mapping of the 3D temperature profiles in the respective container, and understanding of its F/T geometry. The set-up further helps to identify a sub-set of the initial multiple temperature probe positions.

In the following case study, we exemplify this approach for 2 L DS bottles frozen to −80 °C. The initial test set-up for temperature mapping experiments (Figure 1a) consisted of eight temperature probes, four along the edge axis (in the corner of the rectangular bottle) and four along the center axis.

**Figure 2:**
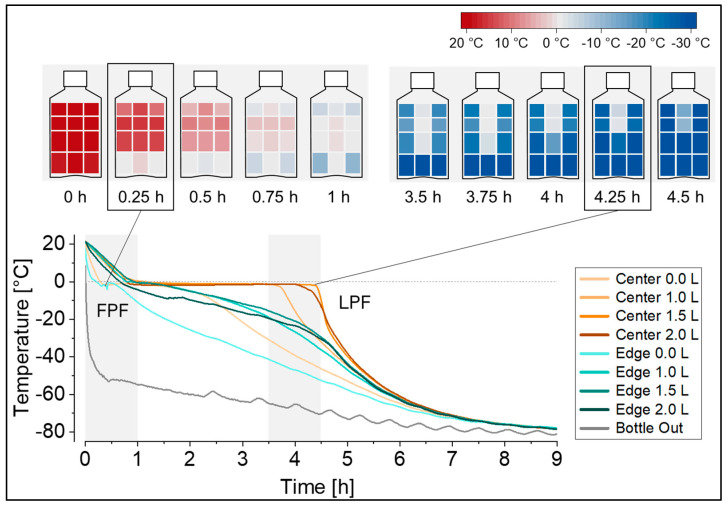
Temperature profiles during freezing at −80 °C recorded at eight temperature probe positions in the 2 L DS bottle filled with 2 L of surrogate solution. The freezing geometry is visualized as heat maps for the gray time spans in the temperature profiles, as well as for the first point to freeze (FPF) and the last point to freeze (LPF) in the bottle. Heat maps were generated from the same dataset and edge positions were mirrored for visualization purposes.

In this set-up, the temperature profiles at the defined measuring positions revealed that freezing started at the bottom edge (FPF location) and continued in a predominantly radial freezing geometry towards the upper radial center, with the LPF located below the liquid surface, close to the fill level indicator height of 1.5 L (Figure 2). The freezing geometry can be visualized as heat maps at selected time points in the freezing profile, as also shown in Figure 2, or alternatively as contour plots. The resolution of these temperature mappings can be further increased by the addition of more temperature probes, e.g., at specific regions of interest like in the upper radial center at the expected LPF location. Notably, freezing in a 5 L DS bottle (filled with 5 L surrogate solution, and equipped with a total of 12 temperature probes instead) revealed a comparable freezing geometry, and showed the LPF located below the liquid surface, in fact, close to at a fill level indicator height of 4.0 L.

Temperature profiles, as displayed in Figure 2, are characterized by an initial cooling phase followed by ice nucleation at the FPF indicated by the characteristic temperature rise from the supercooled solution temperature to the freezing point of the solution. Ice nucleation is followed by a plateau phase with propagating ice formation (exothermal crystallization of water), and subsequent cooling to the target freezing temperature by passing the glass transition temperature (or eutectic temperature, depending on the solution) until reaching the LPF in the container. Notably, the cooling rate at the end of the plateau phase at a distinct position in the bottle, indicated by the steepness of the curve, differs substantially between positions. While the FPF position experienced the slowest cooling rate, the LPF position experienced the fastest cooling rate. For the latter, this is because ice crystallization is complete and no more latent heat is released into the system. Determination of the stress time between the onset of nucleation at the FPF (edge 0.0 L) until reaching the glass transition of the solution (−34 °C) at the LPF (center 1.5 L) yielded a stress time of 4.5 h. If previous technical set-ups with only one measuring position—typically at the LPF—would have been used, a stress time of only 3.8 h would have been measured in this configuration, underestimating the stress time by approximately 15%. Also, the exact time point of nucleation at the LPF position (center 1.5 L) would have to be assumed, for example, when reaching a temperature below 0 °C, since the characteristic step jump indicating the onset of nucleation cannot be detected at later freezing positions.

**Figure 3:**
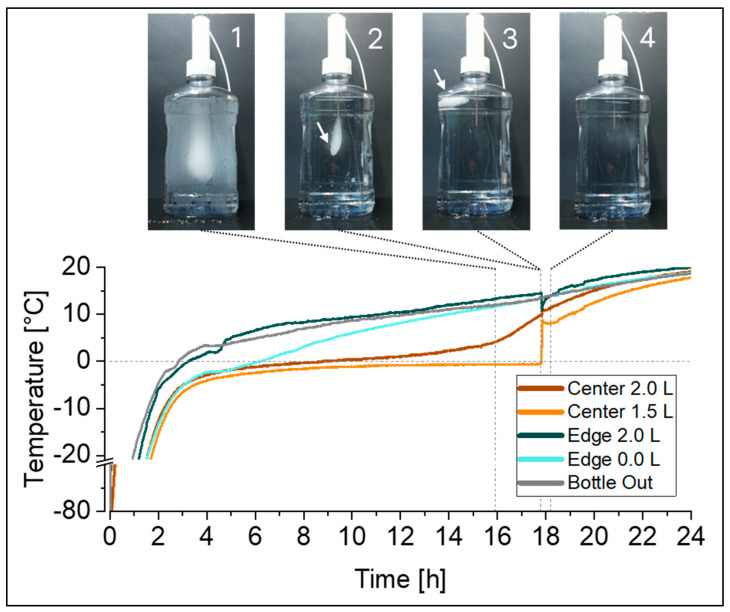
Time-lapse monitoring of a 2 L bottle during thawing from −80 °C at RT was correlated with the simultaneously recorded temperature profiles using the optimized temperature mapping set-up (Figure 1b). Pictures show the melting ice block when it is still attached to the temperature probes in the center (1, 2), the last piece of ice floating to the top after detachment from the temperature probes (3), and the time point at which last piece of ice completely disappeared (4). Arrows (2, 3) are presented to guide the eye to the melting ice block.

For thawing, the temperature profiles recorded at the eight temperature probe positions in the 2 L DS bottles (Figure 1a) identified the FPT in the top edge, and the apparent LPT in the center, at liquid level indicators close to 1 L and 1.5 L. However, given the fact that the last piece of ice will float on top, the LPT location is also expected to be at the liquid surface. In test set-ups that include temperature probes inside the liquid, as studied in this work, the ice is to some extent hindered from floating due to its attachment to a temperature probe (Figure 3, picture 2). At a certain point in time, the last piece of ice detaches from the temperature probe and floats to the surface of the solution (Figure 3, picture 3). The presence of the remaining floating ice will not be captured by the temperature probes, and therefore the actual time point of the LPT and its location can be elucidated by visual monitoring only (see also Figure S2 in Supplementary Materials).

As demonstrated in our example, the time gap between the detachment of the ice (Figure 3, between pictures 2 and 3) and the actual time point at which the ice completely disappeared (Figure 3, picture 4) was rather short. In this set-up it was <2.2% of the total thawing time; however, this may vary between set-ups (e.g., container size, fill level, and thawing process).

As thawing times can only be accurately determined visually, we recommend to visually verify the LPT in addition to the temperature monitoring, to identify the actual time point of complete thawing. We further propose a camera-based monitoring set-up, as described in detail above, providing a convenient solution for detecting this point in time and avoiding the need for an operator to periodically check or be present during the entire process.

As a result of the initial temperature mapping, an optimized set-up was defined, with four temperature probe positions covering only the relevant positions (Figure 1b), which are in general—but this may vary depending on the scope of data analysis—the FPF, LPF, and the FPT positions, as well as the apparent LPT position (equal to the LPF position in this set-up). The experimentally informed positioning of the temperature probes in combination with the camera-assisted determination of the LPT to evaluate the F/T process presents a simplified test set-up enabling efficient and specific F/T process characterization in a defined container configuration.

### 3.2. Liquid Sampling

**Figure 4:**
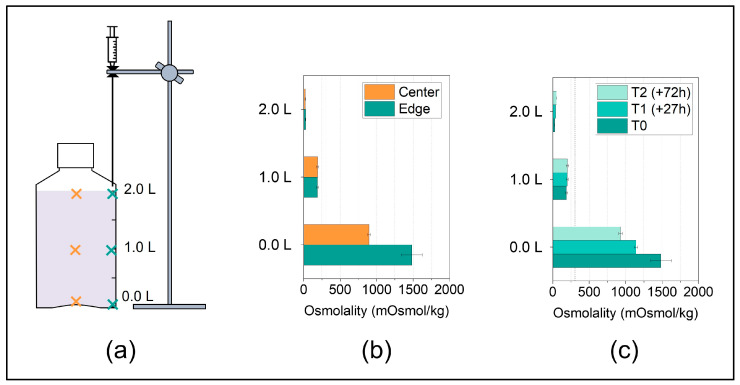
(**a**) Sampling positions after thawing at three fill level indicator heights i.e., at 0.0 L, 1.0 L, 2.0 L, along the center axis (orange crosses) or along the edge axis (cyan crosses). (**b**) Concentration gradients of a surrogate solution in 2 L DS bottles after thawing from −40 °C to RT quantified by osmolality in liquid samples from the edge and center positions and (**c**) at different time points during thawing for the edge positions. The data are presented as mean ± standard deviation of two independent experiments. The accentuated dashed line at 306 mOsmol/kg represents the initial osmolality of the surrogate solution.

Sampling of the thawed solution is typically performed to analyze concentration gradients or stability-indicating parameters at different sample locations after exposure to the F/T processes. In most previous studies, the sampling positions and the number of samples collected varied, and also the sampling procedures used are usually not described in much detail. A very recent study by Blümel et al. [24] describes a sampling procedure for 2 L DS bottles using Pasteur pipettes, which means that sampling positions are selected manually increasing method variability. The herein described sampling procedure presents an optimized set-up due to the lab stand-aided sampling with fixed positions (Figure 4a). This implies less variation in the measurement position and particularly avoids the mixing of the solution during sampling. This is because in our optimized set-up, it is only necessary to enter the solution once with the sampling needle to collect multiple samples along an axial position, which is enabled by the use of a syringe valve.

The following case study applies the optimized sampling procedure. We chose six sampling positions at two horizonal positions (Figure 4a) in order to map the vertical concentration gradients, which are expected to form after thawing, as reported in previous studies [4,24,28].

In line with the literature, the concentration gradients in the surrogate solution after thawing in the 2 L DS bottle from −40 °C to RT analyzed by osmolality revealed a pronounced vertical concentration gradient (Figure 4b). In addition, in the bottom region, the concentrations in the edge were significantly higher compared to the ones in the center. This is likely due to the concave-shaped bottom of the bottle as gravitation drives sedimentation of high-concentrated fraction, leading to the accumulation of a highly concentrated solution at the lowest points in the container, which for these PharmaTainer^TM^ bottles are the edges. At the top of the liquid level, such pronounced concentration differences were not observed between the horizontal positions in line with previous reports by Blümel et al. [24].

In a last step, we studied the changes in the vertical concentration gradients depending on time after thawing without additional, active mixing. The concentration gradients decreased over hours/days but were still pronounced after three days (Figure 4c). When comparing different thawing procedures or protocols, we recommend taking samples as close as possible after complete absence of ice to capture the worst-case concentration gradient, even though diffusion after thawing has been shown to only slowly cause homogenization within the segregated solution [24].

In summary, for PharmaTainer^TM^ bottles, the sampling positions at the top and at the bottom edge represented the positions of lowest and highest concentrations, respectively.

## 4. Conclusions

The presented methodologies provide optimized test set-ups for studying F/T processes in PharmaTainer^TM^ bottles by temperature mapping and sampling of the thawed solution. We report the experimentally justified selection of measuring and sampling positions such as for the FPF, LPF, and LPT enabling the accurate determination of relevant time spans (e.g., stress time) and evaluation of concentration gradients. We further highlight the camera-assisted inspection of the thawing process to determine the true LPT position and actual thawing time. In particular, we provide a detailed description of the technical implementation of the measuring set-up, constituting a robust (fixed measuring positions), easy-to-implement, time- and sample-conscious methodology for F/T process characterization in DS bottles, which has the potential to be applied to a variety of DS containers.

## Data Availability

The original contributions presented in the study are included in the article and Supplementary Materials, further inquiries can be directed to the corresponding author.

## Supplementary Materials

The following supporting information can be downloaded at: https://www.mdpi.com/article/10.3390/mps7050068/s1, Figure S1: Variability of temperature profiles. Figure S2: Pictures of thawing process in 2 L DS bottles with and without temperature monitoring set-up.
