# Supplementary material for: Optimization of Methodologies to Study Freeze/Thaw Processes in Drug Substance Bottles
Source: Methods Protoc. 2024 Sep 4;7(5):68. doi: 10.3390/mps7050068 (PMC11417747; doi:10.3390/mps7050068)
Supplement: Supplementary file 1 [file mps-07-00068-s001.zip › mps-3103340-supplementary.pdf]

Supplement to

Optimization of Methodologies to Study Freeze/ Thaw Processes in Drug Substance Bottles

Variability of temperature profiles

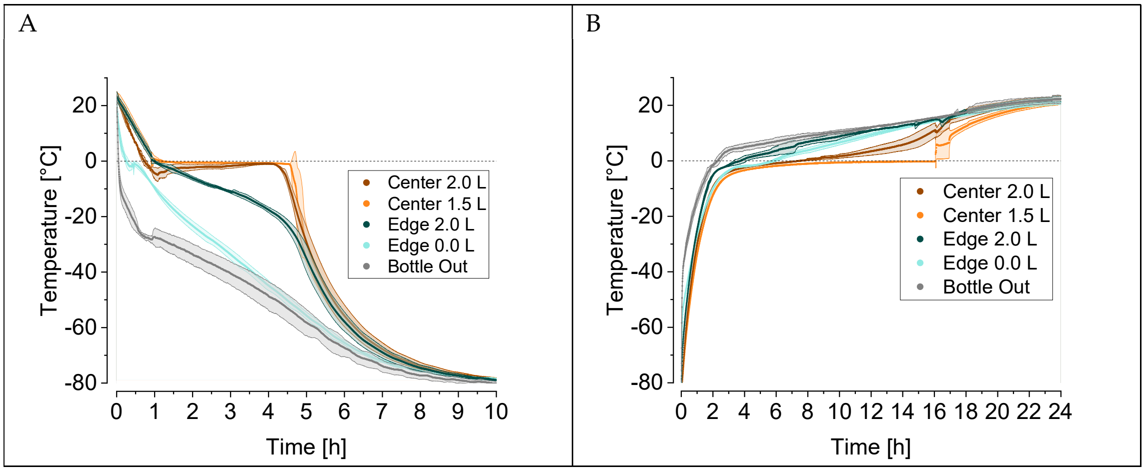

Figure S1: *Variability of temperature profiles. Temperature profiles at four selected positions in the 2L DS bottle, as well as outside of the 2L DS bottle (as indicated in legends and shown in Figure 1b in the manuscript) were recorded for three experimental runs during (A) freezing at -80°C and (B) subsequent thawing at RT. Data is presented as means (solid line) and standard deviations (transparent band) of the three experiments.*

The temperature mapping set-up showed low variability, in particular at the four selected positions representing the FPF, LPF, FPT, and LPT. A slightly higher variability can be observed during thawing (Figure S1B) at the apparent LPT (Center 1.5 L), which is due to the detachment of ice from the temperature probe described in detail in the manuscript.

Pictures of thawing process in 2 L DS bottles

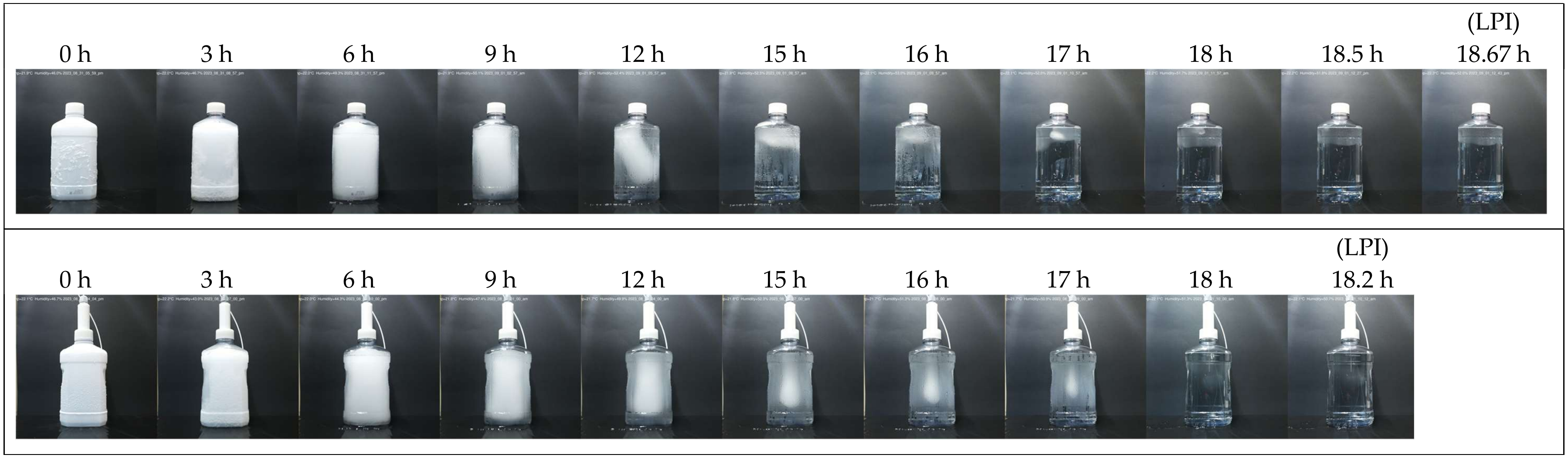

Figure S2: *Thawing of a 2 L DS bottle filled with 2 L of surrogate solution without (above) and with (below) the temperature probe set up until the time point at which the last piece of ice (LPI) disappeared.*

We tested the impact of the temperature probes on the thawing process and compared bottles with and without temperature probes by time lapse monitoring. The temperature probes in the center hold the ice block in an upright position in place, while without temperature probes the ice block at certain point tilts and starts to float at the surface. The impact on the overall thawing time due to conduction of the temperature probes was considered negligible in this case, in fact, thawing with temperature probe was about 30 min faster compared to without temperature probes.
